# Catalyzing action on HIV/SRH integration: lessons from Kenya, Malawi, and Zimbabwe to spur investment

**DOI:** 10.1080/16549716.2022.2029335

**Published:** 2022-03-24

**Authors:** Janet Fleischman, Fannie Kachale, Fatima Mhuriro, Mary Mugambi, Getrude Ncube, Albert Ndwiga, Rose Nyirenda, Anna Carter, Jessica Rodrigues, Kate Segal

**Affiliations:** a Center for Innovation in Global Health, Georgetown University Medical Center, Washington, DC, USA; b Department of Reproductive Health, Ministry of Health, Lilongwe, Malawi; c Reproductive Health Unit, Ministry of Health and Child Care, Harare, Zimbabwe; d NASCOP, Ministry of Health, Nairobi, Kenya; e Department of AIDS/TB, Ministry of Health and Child Care, Harare, Zimbabwe; f Department of Family Health, Ministry of Health, Nairobi, Kenya; g Department of HIV/AIDS, Ministry of Health, Lilongwe, Malawi; h AVAC, New York, NY, USA

**Keywords:** HIV, sexual and reproductive health, gender issues, COVID-19

## Abstract

The HIV pandemic has long revealed the inequities and fault lines in societies, one of the most tenacious being the pandemic’s disproportionate impact on adolescent girls and young women. In east and southern Africa, renewed global action is needed to invigorate an effective yet undervalued approach to expanding HIV prevention and improving women’s health: integration of quality HIV and sexual and reproductive health (SRH) services. The urgency of advancing effective integration of these services has never been clearer or more pressing. In this piece, national health officials from Kenya, Malawi, and Zimbabwe and global health professionals have joined together in a call to catalyze actions by development partners in support of national strategies to integrate HIV and SRH information and services. This agenda is especially vital now because these adolescent girls and young women are falling through the cracks due to the cascading effects of COVID-19 and disruptions in both SRH and HIV services. In addition, the scale-up of pre-exposure prophylaxis (PrEP) has been anemic for this population. Examining the opportunities and challenges of HIV/SRH integration implemented recently in three countries – Kenya, Malawi, and Zimbabwe – provides lessons to spur integration and investments there and in other nations in the region, aimed at improving health outcomes for adolescent girls and young women and curbing the global HIV epidemic. While gaps remain between strong national integration policies and program implementation, the experiences of these countries show opportunities for expanded, quality integration. This commentary draws on a longer comparative analysis of findings from rapid landscaping analyses in Kenya, Malawi, and Zimbabwe, which highlighted cross-country trends and context-specific realities around HIV/SRH integration.

## Background

The HIV pandemic has long revealed the inequities and fault lines in societies, one of the most tenacious being its disproportionate impact on adolescent girls and young women [1]. In east and southern Africa, where most HIV infections result from heterosexual transmission [2], renewed global action is needed to invigorate an effective yet undervalued approach to expanding HIV prevention and improving women’s health: integration of quality HIV and sexual and reproductive health (SRH) services, defined as joining together different HIV and SRH services or programs to maximize improved outcomes [3].

Despite broad recognition that integrated HIV and SRH services are more aligned with women’s and girls’ preferences and lifestyles and can lead to improved health outcomes, progress has been undercut by fragmented, siloed funding streams from development partners [4], limited implementation at the country level, and a failure to engage young women in HIV prevention efforts and SRH services that can meet their needs [5].

The urgency of advancing effective integration of these services has never been more clear or more pressing. This is why we have come together – national health officials from Kenya, Malawi, and Zimbabwe, and global health professionals – in a call to catalyze actions by development partners in support of national strategies to integrate HIV and SRH information and services [6]. Data across countries show higher rates of unmet need for contraception and HIV incidence among adolescent girls and young women, as well as other HIV and SRH indicators that merit attention (see Table 1). This agenda is especially vital now because these adolescent girls and young women are falling through the cracks due to the cascading effects of COVID-19 and disruptions in both SRH and HIV services [7,8]. In addition, the scale up of pre-exposure prophylaxis (PrEP), a highly effective biomedical HIV prevention tool, has been anemic for this population [9].

**Table 1.** Comparison of demographic and epidemiological data in Kenya, Malawi and Zimbabwe

Metric	Kenya	Malawi	Zimbabwe
HIV prevalence rate (female, 15–24) (2020)^1^	2.1%	3.7%	5.2%
HIV incidence rate (female, 15–24) (2020)^1^	1.9%	3.1%	4.2%
Progress toward 90–90-90 among females age 15 +^1^	98–92-94	94–95-91	96–98-95
Unmet need for FP among women in marriage/union (15–49)^2^	12%	13%	8%
Unmet need for FP among women in marriage/union (15–19)^3^	23%	24.9%	12.6%
Modern contraceptive prevalence rate (all women)^4^	45%	48%	49%
Injectable (% of method mix)	47.9%^5^	49.8%^6^	15.1%^7^
Oral contraceptive pill (OCP) (% of method mix)	14.1%^5^	3.8%^6^	56.5%^7^
Year oral PrEP introduced	2015	2017	2016
Oral PrEP initiations (Q3 2021, all populations)^8^	127,904	3,853	48,738
Married by age 18 (female)^9^	23%	42%	34%
Percentage of 20–24 year-olds who gave birth before age 18^3^	23.3%	34.7%	22%
Enrolled in secondary school (female)^4^	46%	35%	49%
HIV testing among pregnant women (2020)^1^	85%	98%	86%
Coverage of pregnant women who receive ART for PMTCT (2020)^1^	94%	98%	87%
Gender Inequality Index (out of 189)^10^	126	142	129

^1^UNAIDS. AIDSinfo – Global factsheets 2020 [cited 9 December 2021]. Available from: https://aidsinfo.unaids.org/

^2^UNFPA. World population dashboard [cited 9 December 2021]. Available from: https://www.unfpa.org/data/world-population-dashboard

^3^UNFPA. Adolescents and youth dashboard. 2020 [cited 9 December 2021]. Available from: https://www.unfpa.org/data/dashboard/adolescent-youth

^4^UNFPA: State of the World Population 2021 [cited 9 December 2021]. Available from: https://www.unfpa.org/data/dashboard/adolescent-youth

^5^FP2020. Kenya: FP2020 core indicator summary sheet: 2019–2020 annual progress report [cited 9 December 2021]. Available from: https://fp2030.org/sites/default/files/Kenya%202020%20CI%20Handout.pdf

^6^FP2020: Malawi: FP2020 core indicator summary sheet: 2019–2020 annual progress report [cited 9 December 2021]. Available from: https://fp2030.org/sites/default/files/Malawi%202020%20CI%20Handout.pdf

^7^FP2020: Zimbabwe: FP2020 core indicator summary sheet: 2019–2020 annual progress report [cited 9 December 2021]. Available from: https://fp2030.org/sites/default/files/Zimbabwe%202020%20CI%20Handout.pdf

^8^AVAC. The global PrEP tracker. [cited 9 December 2021]. Available from: https://data.prepwatch.org/

^9^UNICEF. Child marriage data [cited 9 December 2021]. Available from: https://data.unicef.org/resources/dataset/child-marriage/

^10^UNDP. Gender inequality index [cited 9 December 2021]. Available from: http://hdr.undp.org/en/composite/GII

## Discussion

Examining the opportunities and challenges of HIV/SRH integration implemented recently in three countries – **Kenya, Malawi, and Zimbabwe** – provides lessons that we hope will spur integration and investments there and in other nations in the region, aimed at improving health outcomes for adolescent girls and young women and curbing the global HIV epidemic. While gaps remain between strong national integration policies and program implementation, the experiences of these countries show opportunities for expanded, quality integration. We propose the following agenda for action:

### Strengthen the policy environment

Kenya, Malawi and Zimbabwe show strong regional leadership when it comes to developing conducive national policies, strategies, and guidance for HIV/SRH integration, even before PrEP was introduced. However, translating these policies into health service delivery and provider practice at the local/district levels continues to present challenges, especially in reaching adolescent girls and young women with youth-friendly services around HIV and SRH. Conducive policies should be accompanied by the resources required to strengthen the capacity of health systems and health providers to operationalize quality integration.

### Integrate service delivery that is youth friendly with community-based options

HIV and SRH services through public sector clinics face significant challenges, including funding, training, commodities, and the provision of youth-friendly health services. These challenges are aggravated by judgmental attitudes of healthcare providers toward sexually active adolescent girls and young women. Kenya [10], Malawi [11], and Zimbabwe [12] recognize the need for integrated and youth-friendly services, but only limited integrated centers exist. Strengthening integrated service delivery in the public and private sectors, through static clinics and mobile outreach and through community-based options, would help increase quality, confidentiality, and access, essential to improving outcomes for adolescent girls and young women. Community-based, de-medicalized delivery of HIV/SRH services is an important avenue to reach this population. This also involves increasing outreach to gatekeepers, including parents, religious, and community leaders, to help change social norms that impact adolescent girls’ and young women’s access to HIV and SRH services.

### Include PrEP in SRH and HIV guidelines and services

With PrEP acknowledged as a key tool for HIV prevention, this is an unprecedented moment to ensure that PrEP for adolescent girls and young women is included in SRH and HIV guidelines and services. Since most women and girls access services through the public sector [13], offering HIV testing and PrEP through these family planning and antenatal clinics can enhance uptake and retention, avoid the stigma associated with HIV services, and provide opportunities for cost sharing. This integrated approach is also essential to prepare for next-generation prevention products that are on the horizon, including the dapivirine vaginal ring, the long-acting injectable, cabotegravir (CAB-LA), and the dual-prevention pill.

### Disaggregate data and incorporate new indicators for adolescent girls and young women

Reaching adolescent girls and young women with HIV and SRH services is also challenging due to the lack of relevant, timely, and useable data to detect risk and enhance targeting and program performance. Improved data to better understand AGYW in all their diversity, including disaggregated by age and sex, combined with integrated program and survey data, are critical for identifying gaps in HIV and SRH service coverage for adolescent girls and young women. Incorporating new indicators to identify at-risk adolescent girls and young women into routine monitoring, such as the presence of STIs, early or unintended pregnancy, and prevalence of gender-based violence, can help target integrated SRH/HIV services and identify where resources are needed to reach these young women.

### Expand demand generation and meaningfully engage young women

Mobilizing demand for HIV and SRH services and for PrEP starts with listening to the preferences and priorities of women, as well as their partners and influencers, and meaningfully engaging adolescent girls and young women in the design and implementation of programs, all of which requires community platforms and interlocutors that they trust and that respond to their needs. Activities to reach girls and women in the three countries – youth or girls clubs, peer education, school-based programs, mobile outreach activities, and radio and ‘edu-tainment’ programs – have demonstrated ways to respond to youth preferences and provide information and to create demand [14]. To ensure quality and impact and to strengthen linkages with and referrals to health facilities, these programs need to be supported and expanded as part of effective integration.

### Address structural barriers through multi-sectoral approaches

Discrimination, lack of economic and educational opportunities, and gender-based violence experienced by adolescent girls and young women are widespread and rooted in harmful gender norms, undermining their sexual and reproductive health and rights and heightening their HIV risks [15]. This calls for concerted efforts to elevate multi-sectoral coordination at the national and local levels and investments in community-driven interventions and advocacy to support women and girls and protect their rights.

## Conclusion

The engagement of HIV donors in HIV/SRH integration for girls and young women, especially the U.S. President’s Emergency Plan for AIDS Relief (PEPFAR) and The Global Fund to Fight AIDS, Tuberculosis, and Malaria, reflects both the opportunities and challenges for expanding integrated approaches. Indeed, effective integration of HIV and SRH services, including through strengthened primary health care, is often undermined by siloed funding from these external partners.

Building on these investments provides a critical opportunity to accelerate HIV/SRH integration and to expand access to PrEP for at-risk girls and young women but will face challenges around often weak support for SRH. To effectively advance this agenda, governments need the support of donors and development partners to build on the momentum around effective HIV/SRH integration and move from strong policy to quality program implementation. Concrete steps could include: establishing district-wide integration programs with monitoring and data collection to track HIV prevention and SRH outcomes; convening national and district-level technical working groups as catalysts for integration, with representation from government HIV and reproductive health departments, implementing partners, civil society, girls and young women, and other stakeholders; leveraging private sector providers and expertise to enhance and expand service delivery and to conduct market segmentation to inform demand creation; and elevating champions for PrEP and for integration – largely composed of girls and young women – to operate across all levels of the health system to promote integrated, youth-friendly services, and to serve meaningful functions at the national, sub-national, facility, and community levels.

The glaring inequities exacerbated by the HIV and COVID-19 pandemics demand that national governments and their partners invest in and reinvigorate integrated approaches to address the needs of girls and women. The experiences of Kenya, Malawi, and Zimbabwe show that this is the time for a fundamental realignment of HIV prevention with sexual and reproductive health and rights to advance health outcomes for women and girls.

## Author contributions

All authors contributed equally to this work.

## Paper context

The HIV pandemic disproportionately impacts adolescent girls and young women, particularly in eastern and southern Africa. Integrating HIV and sexual and reproductive health (SRH) services can expand access to prevention services and improve health. The authors recommend strengthening the policy environment to ensure effective implementation; integrating service delivery with youth and community options; including pre-exposure prophylaxis (PrEP); disaggregating data and incorporating new indicators for girls and young women; expanding demand generation; and addressing structural barriers.
